# Single-Molecule Tethered Particle Motion: Stepwise Analyses of Site-Specific DNA Recombination

**DOI:** 10.3390/mi9050216

**Published:** 2018-05-03

**Authors:** Hsiu-Fang Fan, Chien-Hui Ma, Makkuni Jayaram

**Affiliations:** 1 Biophotonics and Molecular Imaging Center, Department of Life Sciences and Institute of Genome Sciences, National Yang-Ming University, Taipei 112, Taiwan; 2 Institute of Cellular and Organismic Biology, Academia Sinica, Taipei 115, Taiwan; 3 Department of Molecular Biosciences, University of Texas at Austin, Austin, TX 78712, USA; chienhuima@austin.utexas.edu

**Keywords:** single molecule analysis, tethered particle motion, site-specific recombination, serine recombinases, tyrosine recombinases, Cre, Flp, ϕC31integrase

## Abstract

Tethered particle motion/microscopy (TPM) is a biophysical tool used to analyze changes in the effective length of a polymer, tethered at one end, under changing conditions. The tether length is measured indirectly by recording the Brownian motion amplitude of a bead attached to the other end. In the biological realm, DNA, whose interactions with proteins are often accompanied by apparent or real changes in length, has almost exclusively been the subject of TPM studies. TPM has been employed to study DNA bending, looping and wrapping, DNA compaction, high-order DNA–protein assembly, and protein translocation along DNA. Our TPM analyses have focused on tyrosine and serine site-specific recombinases. Their pre-chemical interactions with DNA cause reversible changes in DNA length, detectable by TPM. The chemical steps of recombination, depending on the substrate and the type of recombinase, may result in a permanent length change. Single molecule TPM time traces provide thermodynamic and kinetic information on each step of the recombination pathway. They reveal how mechanistically related recombinases may differ in their early commitment to recombination, reversibility of individual steps, and in the rate-limiting step of the reaction. They shed light on the pre-chemical roles of catalytic residues, and on the mechanisms by which accessory proteins regulate recombination directionality.

## 1. Introduction

Tethered particle motion (TPM) analysis is a relatively simple and affordable biophysical method for following the dynamics of long polymers in solution, and changes in these dynamics with changes in environment or as a result of their interaction with ligands. The output from a TPM experiment is an indirect readout of the mean length of a polymer in conjunction with its persistence length under a given set of conditions, and changes in these parameters with changes in the conditions. In the biological context, TPM has been most commonly used to characterize the interactions of enzyme and non-enzyme proteins with nucleic acids—almost exclusively DNA [1,2,3]. A rare example of the application of TPM to RNA is in characterizing the changes in conformational dynamics and compaction induced by Mg^++^ ions [4]. The purpose of this review is to provide a brief general introduction to TPM, followed by a summary of its application towards gaining mechanistic insights into the action of two distinct classes of DNA site-specific recombinases.

## 2. TPM as a Probe for Polymer–Ligand Interactions

### 2.1. The Experimental Set Up for TPM

**Figure 1:**
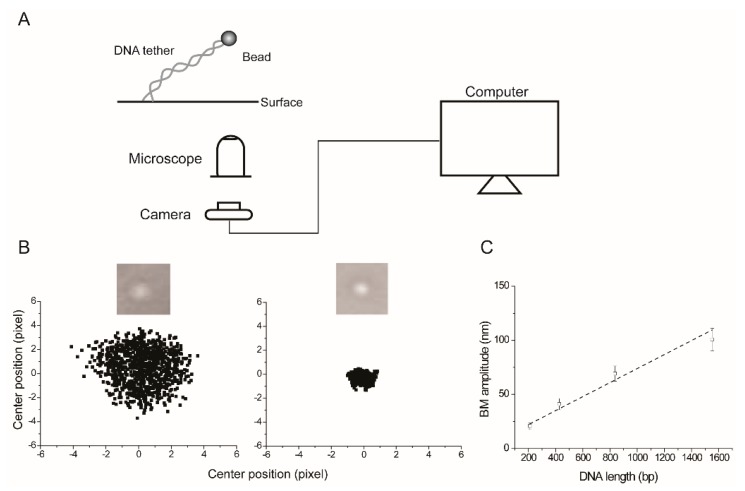
The basic set up for tethered particle motion (TPM) observation and data analysis. (**A**) The polymer being studied (double-stranded DNA in the vast majority of cases) is attached to a planar surface (glass coverslip). Generally, the attachment is mediated by the interaction between digoxigenin placed at one end of the DNA and an antibody to digoxigenin, with which the coverslip is coated. The other end of the DNA tagged by biotin is attached to a bead (generally polystyrene; ~200 nm in diameter) coated with streptavidin. The dynamics of the bead are observed and recorded by an optical microscope fitted with a camera, and connected to a computer. (**B**) The cumulative digitized bead images (from 250 frames; 33 ms for each frame) obtained in two of our experiments with two different DNA molecules are shown here to illustrate the proportionality between the range of bead movement and DNA length. The bead at the right is attached to the shorter DNA. For each frame, the center position of the bead is determined by fitting to a 2D Gaussian distribution. The scatter plots correspond to center positions for bead trajectories over a total of 1000 frames. The Brownian motion (BM) amplitude is estimated as the averaged standard deviation of the center positions along the *x* and *y* axes in consecutive 40-frame windows, with a time resolution of 1.32 s per window. (**C**) The estimated BM amplitudes from our experiments plotted here show their empirical relationship (approximately linear) with DNA lengths within the range of a few hundred to a few thousand bp. In the calibration curve, BM amplitude ‘A’ varies with the tether length ‘L’ in bp as A = (0.0648 × L) + 9.0683. The y-intercept is not constrained to be zero when the tether length is zero. An earlier detailed analysis suggests that the root mean square (RMS) bead displacement varies non-linearly with DNA length [5,8]. Here, the square root of the summed variances from the mean position along both the *x* and *y* directions is computed to obtain RMS*t*. For our purposes, a linear approximation works well. The point to note is that the correlation between bead movement and tether length underscores the utility of TPM as a simple, but effective probe for ligand interactions or biochemical reactions that result in reversible or permanent changes in DNA length.

The first step in a TPM assay is to attach one end of a polymer to a fixed surface, and the other end to a metallic (usually gold) or polystyrene bead. In the case of DNA molecules, these attachments are generally established by labeling the 5′-ends of the two strands with digoxigenin and biotin, respectively, and by coating the tethering surface with anti-digoxigenin and the bead with streptavidin. The polymer and the bead are contained in an aqueous environment, and the Brownian movement of the bead—which is restricted by the length of the polymer tether and its persistence length—is followed by a light microscope fitted with a standard or CCD camera (Figure 1). The bead size can vary rather widely—from ~40 nm (diameter) gold beads to ~1000 nm polystyrene beads—depending on the particular conditions of the experiment at hand. An advantage of the smaller-sized metallic beads is that faster time resolution can be achieved through their use. Measurements using beads of ~200 to ~1000 nm in diameter, and DNA molecules in the length range of ~200 to ~3000 bp, revealed that the minimum time window required to obtain accurate root mean square (RMS) estimates of bead movements increases with bead size [5]. The RMS displacement was estimated as the square root of the sum of the variances of drift-corrected bead positions along the *x* and *y* axes. The experimentally observed values were ~5 s for the smallest bead, and ~20 s for the largest bead. Furthermore, by using smaller beads, distortions in the conformational distributions of the tether due to hydrodynamic and surface effects can be reduced. With larger beads, such effects can be quite significant. The drawback, however, is the weaker intensity of the scattering signal from smaller beads, which may be overcome by dark-field imaging of bead movements or by fluorescence imaging of fluorescently labeled beads. The excursions of the bead in the *x*–*y* plane, averaged over a given time interval, gives its mean Brownian motion amplitude (BM amplitude), which is a function of the tether length. The BM amplitude may be expressed in terms of the RMS bead displacement (as indicated above, along the *x* and *y* directions), or in terms of the mean standard deviation of the bead centroids along the *x* and *y* directions from a sufficient number of consecutive frames ‘*n*’ (as we have done in our experiments; *n* = 40 in Figure 1C). Thus, TPM is well suited to follow physical interactions or chemical reactions that alter the length of the polymer in question. Image analysis and BM amplitude derivations can be performed using commercial software, public resource software, custom written software, or combinations of these. The following URLs provide a sampling of the available analytical tools: https://github.com/gelles-brandeis/Glimpse/wiki; https://imagej.net/Particle_Tracker; http://www.physics.emory.edu/faculty/finzi/research/code.shtml (the Finzi Laboratory, Emory College of Arts and Sciences).

Further details of the theoretical and experimental aspects of TPM may be found in a number of helpful articles written on this subject [1,2,5,6,7,8,9]. Tether length changes as short as 10 nm (equivalent to approximately three duplex DNA turns) can be detected by TPM with a time resolution of 0.1 s [7]. Furthermore, careful studies have been performed to characterize the effects of bead size, DNA tether length, and entropic forces on the measured TPM signals [10,11,12]. Based on this information, analytical methods have been developed to refine the quality of the raw data collected by TPM [8,13,14]. In this review, our principal focus is on the application of TPM to site-specific DNA recombination, by using BM amplitudes of the bead as a reporter of changes in DNA length induced by the recombinase enzyme—with or without assistance from accessory proteins, depending on the system—at individual steps of the reaction.

### 2.2. Biological Applications of TPM

The first use of TPM for the analysis of a biological system dates back to over 25 years ago, when Shafer, Gelles, Sheetz, and Landick applied this technique to measure the rates of movement of *Escherichia coli* RNA polymerase along individual DNA templates [15]. In their set up, it was the polymerase that was immobilized on the glass surface. The DNA with the attached gold bead became indirectly tethered when bound by the polymerase. The act of transcription progressively shortened the length of the tethered DNA, and correspondingly decreased the BM amplitude of the attached bead. This experiment demonstrated the utility of TPM in studying proteins that translocate along DNA. Over the years, TPM has been utilized to investigate not only directional protein movement—for example, by RNA polymerase or the RecBCD helicase [16,17,18]—on DNA, but also various other aspects of DNA biophysics and DNA–protein interactions, both local and global [19]. Examples include DNA flexibility as well as intrinsic and protein-induced DNA bends [20,21]; DNA loping by repressor proteins and by certain Type II restriction enzymes such as SfiI [22,23,24]; chaperone-mediated DNA wrapping into nucleosomes [25]; DNA wrapping around a non-histone protein wheel—the phage 186 CI repressor [26]; Histone H1-promoted DNA condensation [27]; DNA compaction by nuclear factor-Y [28]; metal ion-promoted RNA condensation [4]; protein–DNA filament formation by the archaeal Alba 1 protein [29]; concentration dependent changes in DNA flexibility introduced by binding of the HU protein—revealed as a bimodality in persistence length [30]; and assembly of high-order DNA–protein complexes exemplified by the IS911 transpososome [31] and by regulatory transcription factors [32].

### 2.3. Technical and Analytical Improvements of Basic TPM

TPM has been combined with low continuous-flow stretching forces (~0.2 pN for ~200 nm polystyrene beads) in the force-tethered particle motion (F-TPM) technique to suppress Brownian motion, and thus obtain improved spatial resolution of the bead position with time [33]. This method is particularly suited to following the linear movement of proteins along DNA. F-TPM was used to refine the translocation rate of RecBCD on double-stranded or single-stranded DNA, and to estimate the change in this rate upon encountering the χ sequence (5′GCTGGTGG3′) [33]. In other experiments, TPM has been combined with considerably higher forces (~0.5 to ~3 pN) applied by optical tweezers [34]. Using these stretch forces, it was possible to demonstrate tension-mediated acceleration of DNA condensation by the Hha/HNS protein complex [34]. Recently, the principle of TPM has been adapted by tethered fluorophore motion (TFM), in which the bead is replaced by a fluorophore [35]. Here, changes in effective DNA length resulting from relatively large-scale motions—and reported by the diffusional freedom of the fluorophore—are monitored using a fluorescence microscope and standard fluorescence techniques and analytical tools. TFM can be combined with fluorescence energy transfer (FRET) to simultaneously follow both small-scale and large-scale conformational transitions in DNA. Another promising advance is a multiplex TPM strategy for high-throughput experiments to study a variety of DNA–protein complexes, including the DNA–gyrase complex and the transcriptionally active DNA–RNA polymerase I complex [36]. An important innovation in high-throughput TPM has been the fabrication of biochips on which >500 DNA molecules could be simultaneously analyzed [37]. A microcontact printing process was used to produce nanoscale arrays on which individual DNA molecules could be positioned in a controlled manner by self-assembly. This method was applied to examine the processivity of T7 exonuclease [37], and to investigate the context-dependent control of site-specific recombination by the FtsK DNA translocase within the bacterial chromosome [38]. A potential new development under progress in Professor Garini’s laboratory (Bar-llan University, Israel) is 3D-tethered particle motion (3D-TPM). In standard TPM, as noted earlier, the bead positons are determined in the lateral (*x*–*y* plane), and follow Gaussian distribution. However, by using total internal reflection (TIR) microscopy, measurement of the axial distribution—which is normally not taken into account—now becomes possible. As the intensity of the evanescent electromagnetic field decreases exponentially with the distance from the surface, the height of the bead with respect to the tether point can be derived from the intensity of light scattered by it. The utility of TIRF (total internal reflection fluorescence) microscopy with synchronous stroboscopic illumination in characterizing the three dimensional behavior of a DNA-tethered bead in time and space was recognized as early as 2005 [10]. The first set of experiments demonstrated the superior quality of DNA looping data by TIRF-TPM by its ability to discriminate authentic DNA loop formation from surface adsorption events. However, the application of the method has been limited, and its potential remains yet to be fully exploited.

### 2.4. TPM Analysis of Site-Specific Recombination

**Figure 2:**
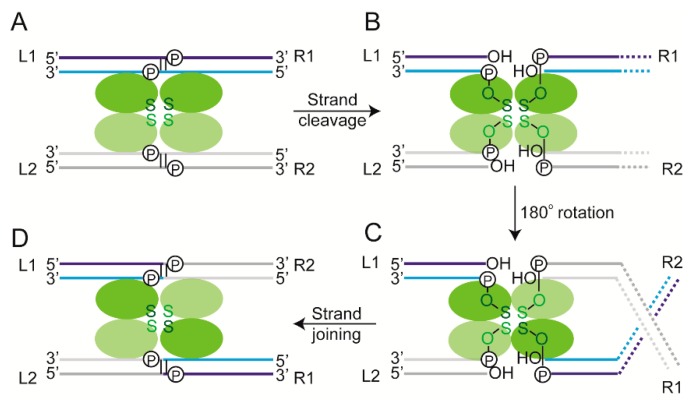
Serine site-specific recombination. (**A**) During serine recombination, the two core target sites (each bound by a recombinase dimer) are brought together in an activated synapse. The dimers bound to the two sites are distinguished by coloring them in different shades of green. The green color indicates the chemically competent state of all four recombinase monomers. In the case of tyrosine recombination (shown in Figure 3), only two of the four recombinase monomers are in the ‘active’ state at a time. The two partner sites are colored differently to better illustrate the DNA rearrangement resulting from recombination. Within a DNA site, the two strands (‘top’ and ‘bottom’) are shaded dark and light, respectively. (**B**) The scissile phosphodiester bonds on the two strands (separated by 2 bp; marked ‘P’) are then cleaved in both partner sites. The recombinase gets attached to one end of the cleaved strand by a 5′-phosphoserine linkage. The adjacent DNA end carries a 3′-hydroxyl group. (**C**) The cleaved complex undergoes a 180° rotation, and is aligned in the recombinant partner configuration. The rotation between the cleavage and joining steps can be appreciated by the DNA crossing introduced as a result. The dashed lines represent DNA flanking the target sites to the right. (**D**) Strand joining by a chemical reversal of the cleavage step completes a round of recombination. In (**A**–**D**), the left to right orientations of the partner sites are indicated as L1-R1 and L2-R2 (before recombination), and as L1-R2 and L2-R1 (after recombination). They are arranged in a parallel fashion, that is, directed the same way in the plane of the synapse. For simplicity, the figure omits accessory sites and the DNA–protein interactions occurring at such sites, which are essential for reaction by some of the serine recombinases. The color schemes for DNA strands and protein subunits are kept the same in Figures 3 and 9, depicting schematics of recombination reactions.

**Figure 3:**
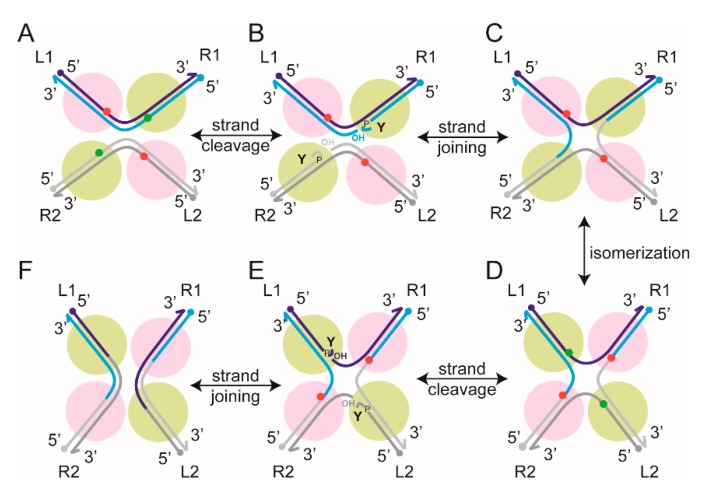
Tyrosine site-specific recombination. (**A**) Within the core recombination synapse, the partner sites (L1-R1 and L2-R2), each bound by two recombinase monomers, are arranged in an anti-parallel geometry. The bound sites have a considerably larger bend than that seen in the sites bound and synapsed by serine recombinases (see Figure 2). The scissile phosphates on the two strands are spaced farther apart (6–8 bp) than in the target sites of serine recombinases. The two scissile phosphates poised for cleavage are shown in green, with the other two in red. The two recombinase monomers responsible for the activation of the phosphates (active monomers) are shown in pale green. The ‘inactive’ ones are shown in magenta. The figure does not include accessory proteins and their interaction with cognate DNA sites required for the activity of a subset of tyrosine recombinases. (**B**) Single-strand cleavage within each partner results in the covalent linkage of the active site tyrosine to the 3′-phopshate, leaving an adjacent 5′-hydroxyl group as its neighbor. (**C**) Strand joining across partners by reversal of the cleavage chemistry produces a Holliday junction intermediate. (**D**) A conformational redisposition of the DNA arms within the synapse (isomerization) activates the scissile phosphates on the strands to be exchanged. The switch between the ‘active’ and ‘inactive’ Flp monomers is indicated by their change in colors (from magenta to pale green, and vice versa). (**E**) As depicted in (**B**), strand cleavage, covalent DNA–protein attachment, and exposure of the 5′-hydroxyl group follow. (**F**) Strand joining completes the duplex exchange to give L1-R2 and L2-R1 as recombinant products. One of the tyrosine recombinases (Cre) that we studied using TPM follows the (**A**–**F**) path strictly, while the other (Flp) differs slightly in the mechanism of strand cleavage. In both cases, a scissile phosphate is activated by the adjacent recombinase monomer (pale green). In Cre, the tyrosine nucleophile for strand cleavage is also provided by the same monomer (in cis); in Flp, it is delivered by a neighboring monomer (magenta; in trans).

Site-specific recombination refers to the breakage and exchange of DNA strands between relatively short, well-defined DNA sequences to produce a new combination of sequences flanking the region of exchange (Figure 2 and Figure 3). The enzymes that carry out these strand breakage/exchange reactions are site-specific recombinases. This review is concerned with ‘conservative site-specific’ recombinases (see below) which are widespread among prokaryotes and mobile DNA elements harbored by them, but are quite rare among eukaryotes [39,40]. They are remarkable for the chemical parsimony with which they bring about diverse, programmed genetic rearrangements resulting in precise physiological consequences. Here, we shall discuss two bacteriophage coded recombinases that determine the choice between lysogeny and lysis, a third phage coded recombinase that promotes stable phage propagation during cell division, and a plasmid coded recombinase that helps maintain the normal steady state plasmid copy number in host cells. Two of the phage recombinases and the plasmid recombinase are members of a mechanistically related family, while the third phage recombinase belongs to a mechanistically distinct family.

We became interested in studying site-specific recombination by TPM, as changes in DNA length associated with the pre-chemical and chemical steps of the reaction make it readily amenable for analysis by this technique. Furthermore, the effective dynamic range of TPM—from several hundred bp to a few thousand bp—is optimal for monitoring recombination in linear DNA substrates. In broad terms, the DNA substrate unbound by the recombinase has the maximum length, observed by TPM as the highest BM amplitude of the attached bead. A molecule experiences stepwise shortening of tether length (a) by the conformational constraints imposed by recombinase binding at the target sites, and (b) by the pairing of sites into a recombination synapse, causing the lowering of BM amplitudes by finite amounts. The shortened tether length of the synapse, as well as the corresponding BM amplitude, becomes permanent if the DNA loop sequestered by the synapse is either trapped as a Holliday junction intermediate or gets deleted as a result of recombination. These aspects will be expanded on when we discuss the behavior of DNA substrates that differ in the relative orientation of the target sites present within them (see below).

When we started our TPM studies on tyrosine recombination [41,42,43,44], there was only one published report on the single molecule analysis of a site-specific recombination system [45], which also utilized TPM. To date, there are a handful of additional publications describing the application of single molecule approaches in the investigation of site-specific recombination [35,46,47,48,49,50,51]. The tools employed include magnetic tweezers, TPM, TFM, TFM-FRET, and PIFE (protein induced fluorescent enhancement). Collectively, these analyses have not only verified many of the mechanistic notions of site-specific recombination derived from ensemble biochemical experiments and structural studies, but also refined them to a new level of resolution. The association of recombinase with targets sites, assembly of synaptic complexes, activation of pre-assembled inactive synapses by protein–protein interactions, conformational changes concomitant with activation, isomerization of DNA intermediates, and rotational movements of DNA during strand exchange could be visualized in real time, and kinetically characterized. It became possible to demarcate rate-limiting steps, and to obtain direct evidence for futile DNA–protein associations, followed by their decay and subsequent successful entry into the normal reaction path. Finally, unsuspected contributions from conserved catalytic residues to the reaction, over and above their roles in directly facilitating the chemistry of phosphodiester breakage and formation, were brought to light.

### 2.5. Recombination Mediated by Conservative Site-Specific Recombinases

Conservative site-specific recombinases belong to two families, namely the serine and tyrosine families—the names indicating the active site nucleophiles utilized by members of each family for the strand cleavage step of recombination [39,40,52,53,54] (Figure 2 and Figure 3). The catalytic serine or tyrosine forms a covalent bond with the phosphate group of the cleaved strand. Chemically, the strand joining step is a reversal of cleavage, and the nucleophile utilized is the hydroxyl group exposed by cleavage. Recombination is the result of strand cleavage within two synapsed target site partners, followed by the joining of the cleaved strands between the partners. The result is the formation of reciprocal recombinant products. Tyrosine recombination is completed in two steps of single strand exchanges, with a Holliday junction as an obligatory intermediate (Figure 3). In contrast, serine recombination is accomplished by concerted double strand cleavage in the DNA partners, rotation of the cleaved DNA, and joining of the strands (Figure 2). In either instance, as strand cleavage and joining utilize transesterification chemistry, there is no exogenous energy requirement and no DNA degradation or synthesis. The phosphodiester bonds are conserved in number between the parental substrate(s) and the recombinant product(s). In general, the recombination chemistry is carried out by four subunits of a recombinase—occasionally, the functional recombinase is comprised of two subunits each of a pair of closely related recombinases—with or without assistance from accessory protein factors. These accessory factors may play architectural or topological roles in the assembly of the recombination synapse, and in doing so, regulate the directionality of the reaction [55,56,57,58].

### 2.6. Steps of Site-Specific Recombination Reported by TPM

**Figure 4:**
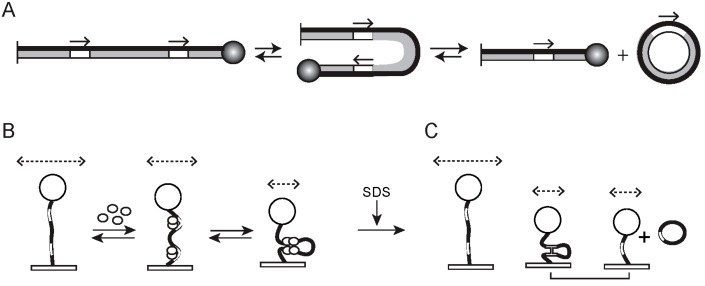
TPM analysis of tyrosine recombination between head-to-tail target sites. (**A**) The linear DNA substrate and the products of the recombination reaction are schematically drawn. The tethering surface and the polystyrene bead are shown as a short vertical line on the left and the sphere on the right, respectively. The relative orientation of the target sites is indicated by the direction of the arrows. (**B**) The changes in length of the DNA tether following the binding of the target sites by the recombinase and at subsequent steps of the recombination pathway are shown schematically. The relative magnitude of the BM amplitudes of the DNA–protein complexes are approximated by the lengths of the dashed, double-headed arrows drawn above the bead. The bound and bent target sites cause an apparent reduction in tether length, which is further accentuated by the synapsis of the bound sites. The exchange of one pair of strands to form the Holliday junction intermediate, or the completion of the excision reaction, will make this length reduction permanent. (**C**) The effect of removing the recombinase from the DNA by SDS addition is schematically illustrated. The non-synapsed molecules, as well as the synapsed molecules that failed to undergo strand exchange, will return to the starting BM amplitude of the DNA substrate, while the Holliday junction and the linear product of excision will retain their low BM amplitude state. The diagram is also applicable to serine recombination, with the following caveats. The conformational distortion or DNA bending introduced by serine recombinases is less prominent than that for tyrosine recombinases. The geometry of the sites within the activated synapse is parallel. As the strands are exchanged by a double strand break-join mechanism, no Holliday junction is formed during the reaction.

**Figure 5:**
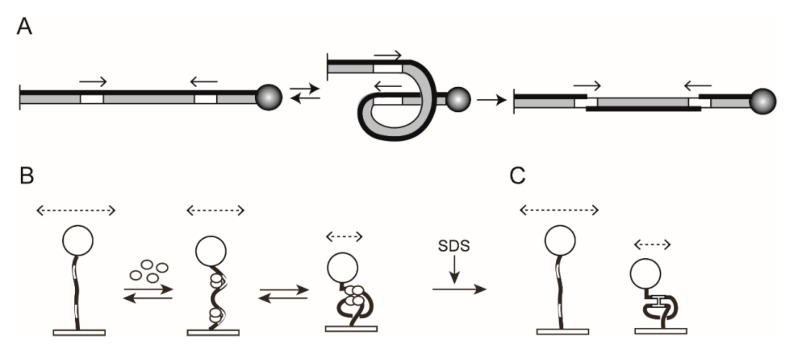
TPM analysis of tyrosine recombination between head-to-head sites. The panels (**A**–**C**) are arranged as in Figure 4. Note that the outcome of the recombination is DNA inversion between the target sites, indicated by the switch in the thickness of the two strands within the inverted segment. The configuration of the looped DNA in synapsed molecules is different for head-to-head and head-to-tail sites in order to accommodate the anti-parallel arrangement of sites. Note that, following SDS addition (**C**), only the Holliday junction intermediate will remain in the low BM amplitude state. Unreacted molecules, as well as molecules that underwent recombination, will be indistinguishable from each other by their BM amplitudes.

As already pointed out, the physico-chemical steps of recombination—binding of the recombinase to each of the two partner targets, synapsis of the partners, and formation of recombinant products during deletion reactions—are accompanied by characteristic changes in the length of DNA, and are ideally suited for analysis by TPM using tethered substrate molecules. During intramolecular recombination, the outcome depends on the relative orientation of the target sites (Figure 4 and Figure 5). When they are head-to-tail (direct orientation), recombination causes the excision (deletion) of the DNA included between the two sites (Figure 4A). When the sites are head-to-head (inverted orientation), recombination results in the inversion of the DNA between the sites (Figure 5A).

The binding of a target site by two subunits of a tyrosine recombinase, or by a dimer of a serine recombinase, is often accompanied by changes in DNA conformation. In the case of tyrosine recombinases, the bound DNA is significantly bent. For one of the systems that we have investigated by TPM [44], the bend angle caused by the interaction of adjacent recombinase monomers on DNA was estimated to be as high as ~140° from gel mobility shift experiments [59,60]. However, the bend angles seen in the crystal structures of the recombination synapses are smaller; 90–100° [61,62]. The difference likely arises from the dynamic nature of the bend. The particular angles captured in the structures might have been selected because of their favorable effects on crystal packing.

TPM is generally sensitive enough to detect the slight shortening of a substrate molecule resulting from protein occupancy of the pair of target sites present within it (Figure 4B and Figure 5B). High-throughput TPM analysis of DNA containing in-phase A_6_-tracts showed that the mean bending angle introduced by one such tract could be reliably estimated as (19 ± 4)° in ~600 bp long molecules attached to 300 nm (diameter) beads [20]. The analytical procedures used here are also capable of accurately deriving significantly higher protein-induced DNA bends—for example, ~160° resulting from the binding of IHF (integration host factor)—from TPM data. In our TPM experiments using a ~1200 bp DNA substrate, we noticed ~15 nm reduction in BM amplitude when one target site is occupied by the aforementioned tyrosine recombinase, and ~23 nm reduction when two such sites separated by ~600 bp are both occupied [44].

Synapsis of the bound sites, with looping of the DNA between them, leads to a larger decrease in tether length (Figure 4B and Figure 5B). For the tyrosine recombinase referred to above, the result is a ~40 nm drop in BM amplitude from that of the unbound substrate DNA. This DNA shortening becomes permanent (or resistant to protein dissociation) when a Holliday junction is formed within the synaptic complex (in the case of tyrosine recombination). The effect is the same for completion of the deletion reaction (by a serine or a tyrosine recombinase). The inversion reaction, by contrast, does not change the length of the DNA.

The pre-chemical steps of recombination (recombinase binding to target sites; synapsis of the bound sites) (Figure 4B and Figure 5B) and the chemical steps of recombination (Holliday junction formation; completion of strand exchange in an excision reaction) can be distinguished during a TPM assay by treatment with SDS (Figure 4C and Figure 5C) at a concentration sufficient to dissociate protein from DNA without affecting DNA linkage to the surface or to the bead. Following SDS addition, synapsed molecules that did not undergo strand exchange will return to the ‘high BM’ amplitude of the unreacted substrate. The molecules that underwent Holliday junction formation will retain the ‘low BM’ amplitude of the synapse (for head-to-tail and head-to-head substrates), as will molecules that completed excision (for head-to-tail sites). The molecules that completed the inversion reaction (head-to-head sites) will be indistinguishable from the substrate by BM amplitude (high).

It is helpful to note that the outcome of recombination (deletion or inversion) is determined by the global orientation of the target sites (head-to-tail or head-to-head), regardless of whether the recombinase carrying out the reaction belongs to the serine or tyrosine family. However, the local geometry of the partner sites within the recombination synapse is independent of site orientation, but is dependent on the type of recombinase acting on them. This synapse geometry for a serine recombinase is opposite to that for a tyrosine recombinase (as illustrated in Figure 2 and Figure 3), and is discussed in further detail separately (see below).

### 2.7. Thermodynamic and Kinetic Features of Individual Steps of Site-Specific Recombination

**Figure 6:**
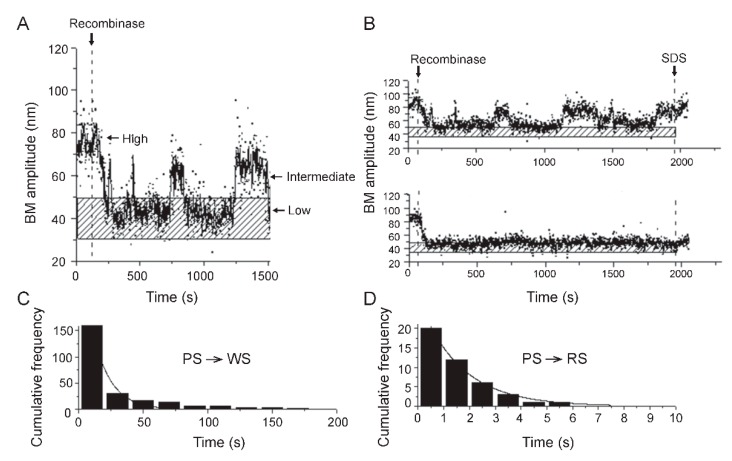
Thermodynamic and kinetic information conveyed by TPM. (**A**) A typical time trace shown here illustrates the BM amplitude changes in a DNA substrate containing a pair of recombination target sites (in head-to-tail orientation) from ‘high’ to ‘intermediate high’ following recombinase occupancy of the sites, and to ‘low’ upon synapsis of the bound sites. (**B**) The top trace is representative of a molecule (with head-to-tail target sites) that bound recombinase, and went through synapsis and synapse dissociation several times without completing recombination (as shown by its return to the high amplitude state after SDS treatment). The bottom trace exemplifies a molecule that completed binding, synapsis, and Holliday junction formation or DNA excision successfully (as signified by the low BM amplitude after SDS treatment). In A and B, the stippled horizontal bar denotes the BM amplitude of the synapsed state. (**C**,**D**) From the dwell times of DNA molecules in the unbound state or as distinct recombinase-bound complexes, kinetic features of the individual steps of recombination can be derived. The histogram plots for the pre-synaptic state (PS) before conversion to wayward synaptic complexes (WS; which are abortive and dissociate), fitted to a single exponential algorithm (**C**), yields a first-order rate constant of (7.2 ± 0.8) × 10^−2^ s^−1^ for this step (PS → WS). From a similar analysis of the transition from pre-synaptic to recombination-competent synaptic complexes (RS; those that promote strand exchange) (**D**), the first-order rate constant is (6.3 ± 0.4) × 10^−1^ s^−1^ (PS → RS). (**A**,**B**) show results from our studies of Flp, a tyrosine recombinase. (**C**,**D**) depict the behavior of ϕC31 integrase (a serine recombinase) in our TPM experiments.

The individual BM amplitude traces recorded in a TPM experiment (Figure 6A,B) reveal the particular state of each DNA molecule in regard to its interaction with the recombinase at any given instant over a certain time course—target sites unbound by the recombinase, target sites occupied by the recombinase, or target sites occupied and synapsed by the recombinase. The occurrence of strand exchange within the synapse is queried by an SDS-challenge at the end of the experiment, in order to see if the BM amplitude of the synapse remains unchanged or shifts to the BM amplitude of the substrate DNA. The time traces also reveal the transition of each molecule between individual states, as well as the life time of a state before its reversal to a prior state or its progression to the next one along the recombination path.

The relative abundance of the different types of DNA–protein complexes, derived from cumulative time traces, gives a thermodynamic picture of the reaction from start to finish, one step at a time. In our work, we have not yet analyzed changes in the amounts of these complexes or in their life times as a function of recombinase concentration or by varying the separation between the recombination target sites. However, analyses along these lines of DNA looping by the lac repressor have been illuminative with respect to the concentration and length dependence of a model transcriptional regulatory system [11]. The existence of at least two distinct looped states were noticed, and their relationship to repressor concentration and the spacing between the repressor binding sites was established. A rather surprising observation was the ability of the repressor, unaided by DNA bending proteins, to form loops when the spacing is shorter than the DNA persistence length. Using a statistical mechanical model, the TPM data could be used to determine the free energy of DNA looping. The power of TPM in understanding the energetic aspects of site-specific recombination systems remains to be fully taken advantage of.

The dwell time histograms for the unbound DNA and the various bound complexes, fitted to an appropriate exponential algorithm, yield the kinetic parameters for each step of the recombination reaction in the forward or reverse direction. As an example, we have shown the life times of two types of recombinase-bound DNA molecules in which synapsis of the target sites follows binding (Figure 6C,D). One subset of the synapses simply dissociates (Figure 6C), while the other subset successfully completes recombination (Figure 6D). The first-order rate constant for each type of synaptic event could be determined by a single exponential fit to the respective plot. The kinetic features of recombination deduced by this type of analysis are elaborated on in the following section (see below).

In sum, TPM is a simple, but highly efficient, method for a complete thermodynamic and kinetic characterization of site-specific recombination. The conclusions reached can be accepted with high confidence when the results from two closely matched DNA substrates, which differ only in target site orientation—head-to-tail in the deletion substrate and head-to-head in the inversion substrate—give mutually concordant results. In our work, we characterized two tyrosine site-specific recombinases (Cre and Flp) and one serine site-specific recombinase ϕC31 integrase [41,42,43,44]. The relevant findings are summarized below.

### 2.8. Insights on Tyrosine Site-Specific Recombination Gained from TPM Studies

The first tyrosine site-specific recombinase to be subjected to a detailed TPM analysis was phage λ integrase (λ-Int) [45] in the ‘excision’ reaction between attL and attR target sites, assisted by the accessory proteins *E. coli* IHF and phage Xis. The protein occupancy of the sites under TPM conditions is too rapid to be measured accurately, giving a lower limit for the first-order rate constant of 3.0 min^−1^. Approximately 50% of the bound sites undergo synapsis, with nearly every one of them completing recombination. Synaptic complexes are stabilized by λ-Int-mediated DNA cleavage before, concomitant with, or following synapsis. The rate-limiting step of recombination occurs post-synapsis, and coincides with, or immediately precedes, Holliday junction formation. The remarkable (~100%) efficiency of the reaction observed in vitro, despite no net free energy change between reactants and products (ΔG = 0), mirrors the *in vivo* situation, where excision, triggered by conditions that place the phage lysogen at a selective disadvantage, is intended to license the lytic pathway with maximum efficiency. Kinetic analyses are consistent with this recombination directionality, presumably conferred by DNA–protein conformational dynamics associated with multiple steps of the reaction.

The findings with λ-Int lays down a useful frame of reference for our studies with Cre and Flp recombinases [41,42,44]. Cre is coded for by phage P1, which is present in the bacterial host as a unit copy plasmid (‘phagemid’). The recombination reaction facilitates equal segregation of phage sisters to daughter cells by resolving genome dimers formed by homologous recombination into monomers [63]. The source of Flp protein is the yeast 2-micron plasmid—a selfish extra-chromosomal DNA element present in the nucleus at a copy number of 40–60 per haploid chromosome content [64]. The physiological role of Flp is in the maintenance of the plasmid copy number by a DNA amplification mechanism triggered by a replication coupled recombination event [65,66]. Recombination inverts one of the bidirectional replication forks with respect to the other, setting in motion iterated copying of the circular plasmid template by two unidirectional forks. The λ-Int, Cre, and Flp paradigms illustrate how evolution has utilized the same chemical mechanism to bring about vastly different physiological consequences under different biological contexts. We studied Cre and Flp under TPM conditions comparable with those used for the λ-Int experiments. Our results underscore general mechanistic similarities, but also reveal subtle contrasts, among these related recombinases.

λ-Int, Cre, and Flp are broadly similar to each other in that the target sites bound by the recombinases—plus IHF and Xis in the case of λ-Int—are robust in undergoing synapsis (~50% to ~90% of the total). The completion of recombination among the synapsed molecules is almost quantitative for λ-Int [45] and Flp [44]. The success rate for Cre is lower, and molecules at the Holliday junction stage are readily detected [41]. Thus, the TPM results show synapsis as a strong early commitment to recombination by λ-Int and Flp, with a lower such commitment on the part of Cre.

The association of Cre and Flp with their respective target sites measured in the TPM assays [41,44] is considerably slower than that reported for λ-Int under comparable conditions [45]. Part of the reason for this striking difference could be the cooperativity in binding provided by IHF and Xis in the case of λ-Int. The measured association rate constants for Cre and Flp (~10^5^ M^−1^ s^−1^) are also significantly smaller than those reported from gel mobility shift analyses (~10^8^ M^−1^ s^−1^ for Cre; ~10^6^ M^−1^ s^−1^ for Flp) [67]. It is possible that the association rates vary with the experimental conditions employed in the two types of assays. The lower sensitivity of TPM in detecting target site occupancy by the recombinase is almost certainly also a contributing factor. Cre and Flp—and tyrosine recombinases in general—are monomers in solution, and bind as monomers to each of the two binding elements of a target site. In our assays, recombinase binding is detected as the reduction in BM amplitude caused by the occupancy of both target sites in a DNA molecule by the recombinase, and the ensuing bending of these sites. Furthermore, stabilization of the DNA bend by interaction between the two recombinase monomers bound to a site may occur with a time delay. In all likelihood, TPM underestimates the rate constants for binding. Ensemble kinetic studies under the same conditions as TPM may help resolve whether the prevailing differences between the two methods arise from experimental conditions or from the limitations of TPM in quantifying recombinase binding to DNA.

**Figure 7:**
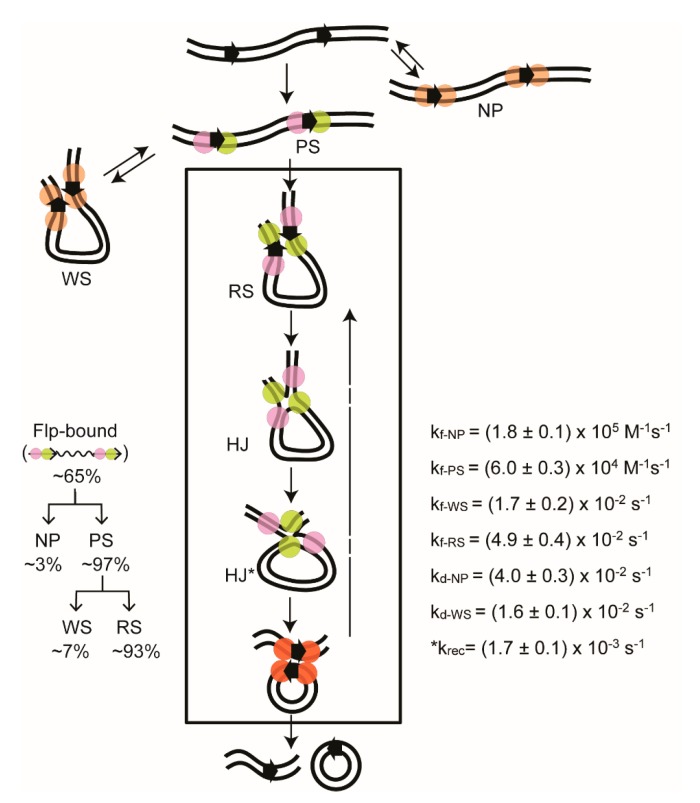
Stepwise view of Flp recombination by TPM. The Flp recombination pathway for the excision reaction using head-to-tail target sites, as inferred from the TPM analysis, is diagrammed. The relative amounts of the individual complexes observed from cumulative single molecule time traces, over a 30 min time course, are indicated. The kinetic constants derived from their life times are listed. The rate constants for the formation and dissociation of a complex is denoted by the subscripts ‘f’ and ‘d’, respectively. The Flp monomers within the recombination synapse are color-coded (pale green or magenta) as in Figure 3 to distinguish between the ‘active’ and ‘inactive’ pairs. All monomers within the product synapse are colored the same, but differently (orange) from those within the recombination synapse, to suggest that the product synapse may have to be reconfigured for initiating the reverse reaction. Similarly, the Flp monomers in the bound complexes that are not in the functional reaction path are shaded in pale orange. The chemical reversibility of the reaction is indicated by the long upward arrow. Rather unexpectedly, our experiments reveal completion of recombination by nearly all of the synapsed molecules. Few Holliday junction molecules are detected. NP = Nonproductive complexes; PS = Pre-synaptic complexes; RS = Recombinogenic synaptic complexes; WS = Wayward synaptic complexes; HJ, HJ* = Holliday junction isomers. The thermodynamic and kinetic features are similar for the inversion reaction from the substrate with head-to-head target sites. The rate constant for completion of recombination in the RS-complexes, k_rec_, is marked by an asterisk to indicate that it was derived from the inversion reaction.

The changes that the bound complexes undergo—both in terms of the amounts of individual complexes and the rates of individual transitions—are reproducibly reported by TPM for Cre and Flp (Figure 7). The initial binding of the target sites produces primarily pre-synaptic complexes (PS-complexes; those that go on to accomplish synapsis), along with smaller amounts of non-productive complexes (NP-complexes; those that dissociate to free substrate without undergoing synapsis). The synapsed molecules can be separated into two classes, ‘authentic’ or ‘recombinogenic’ synaptic complexes’ (RS-complexes) and ‘wayward’ synaptic complexes (WS-complexes). The former class proceeds to perform strand exchange to yield the Holliday junction intermediate or the recombinant products. The synapse is unproductive in the latter class, and dissociates. The abortive complexes (NP- and WS-complexes) are not trapped as dead-end complexes. Following dissociation, they can re-enter the functional reaction path. As the dissociation rates are considerably faster when compared with the overall recombination rate, DNA molecules that formed abortive complexes get more than one attempt at recombination within the time scale of our TPM assays. A stepwise description of the Flp recombination pathway provided by our TPM studies [44] is illustrated in Figure 7, along with the derived kinetic constants.

The rate constant for recombination (conversion of the synaptic complex into recombinant products) cannot be determined directly from the TPM data. For the substrate with head-to-tail sites, a recombination event is revealed only after SDS addition—as an irreversible transition to the low BM amplitude state. Prior to SDS addition, a synapsed molecule in which no strand exchange has occurred, one in which a Holliday junction has been formed, and one that has completed recombination would be indistinguishable from each other because of their identical low BM amplitude. Hence, the precise life time of a synapse before its conversion to recombinant products cannot be determined. We have used the transitions of the DNA molecule containing the head-to-head target sites (inversion substrate) to obtain the rate constant for recombination (k_rec_ in Figure 7) indirectly by the following rationale. Note that, in this case, the parental substrate and the inversion product have the same high BM amplitude. Therefore, the transitions of the low BM amplitude synaptic complexes to the high BM amplitude state must involve two distinct processes. One is the authentic recombination from functionally synapsed complexes (RS → Product; k_rec_) (Figure 7); the other is the dissociation of the abortively synapsed complexes to the substrate (WS → S; k_d-WS_) (Figure 7). A double exponential fit to the dwell times of the synapsed state should give two first-order rate constants, k_rec_ and k_d-WS_. There is no reason to expect the value for k_d-WS_ to be different between head-to-tail and head-to-head sites. In our experiments with Flp [44], one of the estimated rate constants matched that for the head-to-tail sites, and the other was ~10-fold smaller. The latter, (1. 7 ± 0.1) × 10^−3^ s^−1^, was thus inferred to be k_rec_ (Figure 7).

The TPM results from Cre [41] are consistent with the ready reversibility of the strand cleavage and strand joining steps—as expected from the energetics of these reactions, and as evinced by the detection of the Holliday junction intermediate [44]. The junction intermediate is a rare species in the Flp reaction [44]. For Flp, as with λ-Int, the reaction rate appears to be limited at the step of Holliday junction formation or prior to it. In the case of Cre, the rate-limiting step likely occurs after the Holliday junction is formed. While the synaptic complexes formed by λ-Int and Flp are stabilized by strand cleavage, cleavage-dependent stabilization is apparently absent in the Cre synaptic structure.

### 2.9. Reversibility of the Chemical Steps of Recombination and Directionality of the Reaction 

The nearly quantitative conversion of synapsed molecules to recombinants by λ-Int, combined with the kinetic analyses of the recombination steps, has been interpreted to mean that the directionality of the reaction stems from several irreversible steps [45]. The architecture of the recombination complex, arranged with the assistance of IHF and Xis, and the conformational dynamics within it, may provide this irreversibility. Surprisingly, Flp, which acts by itself without help from accessory factors, mimics λ-Int in the directionality of recombination. Perhaps, the conformational changes of the DNA–protein complex accompanying each chemical step pushes the reaction to completion. The reverse reaction may be kinetically impeded if the product synapse has to dissociate and reform—or has to be reconfigured—in a slow step. In the case of recombination between head-to-tail sites, dissociation of the excised circle would also disfavor reversibility.

In the biological context, free reversibility would be a disadvantage, as the forward reaction is designed to bring about a specific physiological effect—phage excision; dimer resolution; or a switch from bidirectional to unidirectional iterative plasmid replication in the case of λ-Int, Cre, and Flp, respectively. The in vitro reversibility of the Cre reaction may not reflect the true attributes of the reaction *in vivo*. It is possible that a high-order DNA organization and/or topology within the bacterial nucleoid may help Cre perform the resolution of phage P1 dimer genomes into monomers in a directed fashion. Furthermore, if dimer resolution is tightly coupled to phage segregation, the probability of reversal—and fusion of monomers—would be minimal. In the Flp case, *in vivo* reversibility would result from a second recombination event, which would negate the effect of the first to restore bidirectional fork movement and terminate plasmid replication. The interval between the two recombination reactions must be under fine regulation in order to maintain a steady state plasmid copy number without large fluctuations.

### 2.10. Characterization of the Active Site Pentad Mutants of Cre and Flp by TPM

The strand cleavage step using the catalytic tyrosine as nucleophile and the strand joining step using the 5′-hydroxyl as nucleophile are assisted by a catalytic pentad conserved among the members of the tyrosine family recombinases [39]. These residues in Cre are Arg-173, Lys-201, His-289, Arg-292, and Trp-315, the corresponding ones in Flp being Arg-191, Lys-223, His-305, Arg-308, and Trp-330, respectively. In addition, an acidic residue—Glu-176 in Cre and Asp-194 in Flp—appears to help the correct spatial positioning of the pentad within the active site [68].

Analyses of the pentad mutants by TPM have revealed previously unsuspected roles played by these residues in the pre-chemical steps of recombination [42]. Furthermore, they reveal important differences between Cre and Flp in the contributions of these residues. Collectively, the pentad residues of Cre enhance the commitment to recombination by kinetically favoring the formation of pre-synaptic complexes. In Flp, these residues perform a similar function by promoting Flp binding to target sites, suppressing the formation of non-productive complexes, and enhancing the rate of synapsis of the recombinase bound sites. The strongest difference between Cre and Flp was observed with respect to Lys-201 (Cre) and Lys-223 (Flp). Lys-223 comes into play at the earliest step of recombinase–target site association, Lys-201 appears to function only after the recombinase-bound sites have been paired by synapsis.

### 2.11. Other Studies on Tyrosine Recombination Using TPM and TPM Related Methods

It should be pointed out that Cre recombination has been also studied using a combination of TFM (which is a conceptual extension of TPM) and TFM-FRET [50]. This approach permitted the observation of individual reaction steps in real time, as well as the analysis of their kinetic properties. A preferred order in the exchange of the two strands of DNA could be demonstrated, and a rate-limiting step following the rapid isomerization of the Holliday junction intermediate could be delineated. The findings suggested the existence of two non-productive synaptic complexes, and revealed an extremely stable product synapse that is refractory to a new round of recombination. TPM or TFM has also been successfully utilized in understanding the mechanistic details of XerCD–dif recombination [47,51], a highly conserved tyrosine recombinase system that coordinates the final steps of bacterial chromosome segregation with cell division. In one study, TPM was used to demonstrate that XerCD–dif recombination synapse is assembled in an inactive form in the presence of the collaborating XerC and XerD recombinases together with a pair of the dif target sites [47]. However, the functional activation of the synapse requires the regulatory DNA translocase FtsK, whose γ-domain increases the rate of synapse formation, in addition to promoting the conversion of the synapse from an inactive to an active state. In the second study, TFM was fortified by FRET and PIFE to follow the long-range, nanoscale conformational dynamics of the recombination reaction modulated by FtsK [51]. Preformed synaptic complexes are activated by their encounter with FtsK and the attendant conformational changes. Following activation, XerD mediates the first strand exchange and Holliday junction formation, to be followed by junction isomerization and then XerC catalyzed junction resolution to recombinant products.

### 2.12. Geometry of Target Sites within the Tyrosine Family Recombination Synapse

The confluence of evidence from structural, topological, and single molecule FRET analyses support an anti-parallel geometry of the DNA partners arranged in an almost perfectly planar fashion within the recombination synapse formed by Cre or Flp [61,69,70,71,72]. Rather unexpectedly, the TPM results provide one more independent piece of evidence for this particular geometry of the recombination synapse.

**Figure 8:**
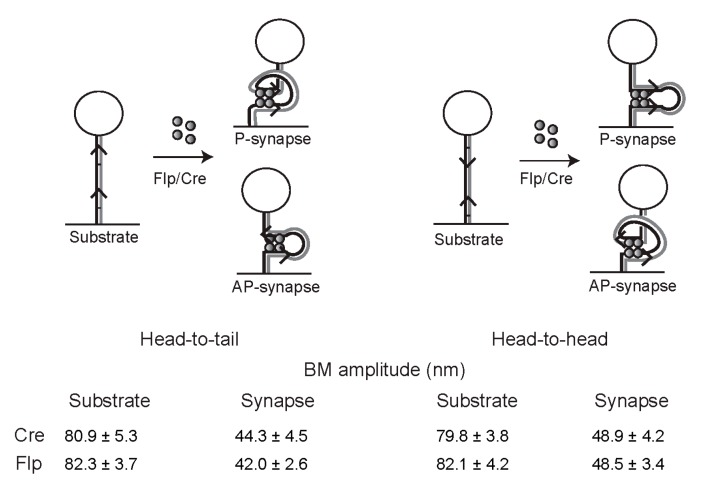
Geometry of target site alignment within the tyrosine recombination synapse inferred from TPM. The schematic diagram illustrates the configurations of the DNA tether when the recombinase-bound sites align themselves in parallel (P) or anti-parallel (AP) geometry within the planar recombination synapse. The head-to-tail (deletion) and head-to-head (inversion) substrates, and their synapsed states, are shown in the left and right panels, respectively. The mean BM amplitudes, tabulated below, are from the TPM analyses of the tyrosine recombinases Cre and Flp. There is a small, but significant increase in the BM amplitude of synapsed head-to-head sites when compared with head-to-tail sites for both Cre and Flp. The higher value is consistent with anti-parallel synapse, as the DNA tether is expected to experience a lower constraint when it enters and exits the synapse from opposite ends, rather than from the same end. This inferred synapse geometry conforms to evidence from other analyses, and is complied within the schematic diagrams in Figure 3, Figure 4 and Figure 5.

The mean BM amplitudes of the synaptic structures formed by Flp were found to be slightly, but reproducibly, different for two DNA substrates containing head-to-tail versus head-to-head target sites, but identical to each other in all other respects [72]. The BM amplitude of the synapse was higher for the head-to-head sites. For these sites, the entry and exit points of the DNA are at the opposite ends of the synapse if the sites conform to anti-parallel geometry (Figure 8). For head-to-tail sites, the same geometry would position the entry and exit points at the same end of the synapse. The constraint on the DNA tether is expected to be slightly higher (shorter effective length; lower BM amplitude) for the proximal, as opposed to the distal, disposition of the entry–exit points. A similar difference in the BM amplitudes of synapsed molecules—higher for head-to-head sites—was also noticed in TPM assays with Cre, suggesting a common geometry of site alignment during tyrosine recombination. The anti-parallel arrangement of the synapse is also consistent with results from the analysis of Cre recombination by TFM and TFM-FRET [50]. Furthermore, similar behavior of wild type Cre and Flp and their catalytically inactive mutants with respect to site orientation and the BM amplitudes of their synapses imply that chemical competence is not a pre-requisite for the selection of the functional site geometry.

### 2.13. TPM Analyses of a Serine Site-Specific Recombinase

The successful application of TPM to shed light on the mechanistic features of tyrosine recombination provided us with the impetus to undertake similar analyses of serine site-specific recombination. The small and large members of the serine family recombinases harbor a characteristic catalytic domain—the SR domain—normally present at the amino-terminus [40]. The SR domain is attached to a carboxyl-terminal domain, which is quite variable in size and properties. The subfamily of small serine recombinases, characterized by a small helix-turn-helix (HTH) DNA binding carboxyl-terminal domain, includes transposon- and plasmid-coded resolvases (resolvases of Tn3 and γδ transposons and Sin recombinase of staphylococcal plasmids, for example) and phage- or bacteria-coded invertases (Gin invertase of phage Mu and Hin invertase of *Salmonella*, for example). There is a wealth of biochemical, topological, and structural information on the mechanism of action of small serine recombinases [40,52,53]. In the subfamily of large serine recombinases, the carboxyl-terminal domain is considerably larger, 300 to 500 amino acids long. A subgroup of the large seine recombinases is comprised of phage-coded serine integrases (the integrases of the *Streptomyces* phage ϕC31, the mycobacteriophage Bxb1, and the *Listeria phage* A118 or prophage LI, for example) [73]. Although the large serine recombinases have not been as extensively characterized as their small counterparts, emerging structural information [74,75], complemented by available biochemical and topological data, is filling this gap.

**Figure 9:**
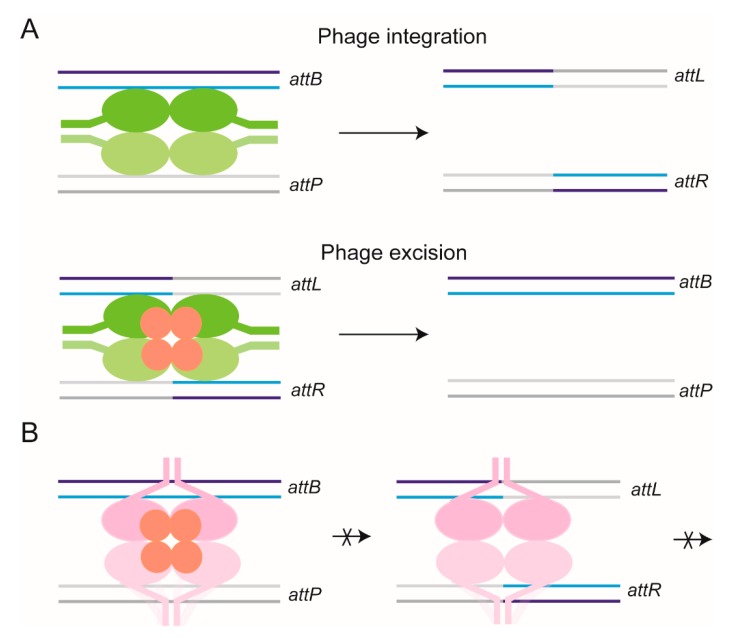
Regulation of directionality during recombination mediated by ϕC31 integrase. In the schematic representations of attP × attB and attL × attR recombination reactions, the active and inactive forms of the integrase dimer are colored green and magenta, respectively. The dimers bound to the two target sites are differentiated by their dark and light shades. (**A**) Integrase forms a functional synapse with attP–attB partner sites, and catalyzes recombination between them. *In vivo*, attP × attB recombination results in the integration of the ϕC31 phage into the host chromosome. The prophage is flanked by attL and attR sites. The attL and attR sites are also targets for recombination by the integrase, assisted by the recombination directionality factor (RDF) protein (colored red). (**B**) The attP × attB reaction is inhibited by RDF, while the attL × attR reaction fails to occur in the absence of RDF. The active and inactive configurations of the synapse appear to be regulated by the interactions of the coiled coil (CC) domains present within the carboxyl-terminal portion of integrase. RDF interaction with the DNA-bound integrase apparently induces alternative synaptic configurations for attL–attR sites (active; **A**) and attP–attB sites (inactive; **B**). The CC domains are drawn as short extensions from the main body of the integrase.

We have chosen the ϕC31 integrase, which catalyzes phage integration and excision, for our TPM studies [43] for several reasons. The attP and attB target sites required for integration and the attL and attR target sites required for excision are relatively short (~50 bp) DNA sequences, and no accessory protein binding sites are involved. The reactions can be performed using linear DNA substrates containing the att sites in head-to-tail orientation or head-to-head orientation to yield deletion or inversion, respectively. These features are in contrast to the stringent topological requirements, as well as selectivity in target site orientation, exhibited by the small serine recombinases, such as Tn3 or γδ resolvase, and the Gin or Hin invertase. The integration reaction (attP × attB) requires only the integrase (four subunits; a pair of integrase dimers), whereas the excision reaction (attL × attR) requires the integrase plus a second phage-coded protein, RDF (recombination directionality factor) (Figure 9). RDF not only promotes attL × attR reaction, but inhibits attP × attB reaction. The system affords the opportunity to address the step(s) at which RDF influences the directionality of recombination. At the time that we initiated the TPM studies of ϕC31 integrase, there was only one reported single molecule analysis of a large serine recombinase [46]—that of the strand exchange mechanism by Bxb1 integrase using a magnetic tweezers set up. In this supercoiling release assay, target site cleavage, DNA rotation, strand joining, and product release were followed in real time. The results were consistent with the relative rotation of the two halves of the cleaved synaptic complex (as diagrammed in Figure 2). An unexpected observation was the long rotationally open state of the synapse, lasting for minutes.

### 2.14. Stepwise Dissection of ϕC31 Integrase Mechanism

**Figure 10:**
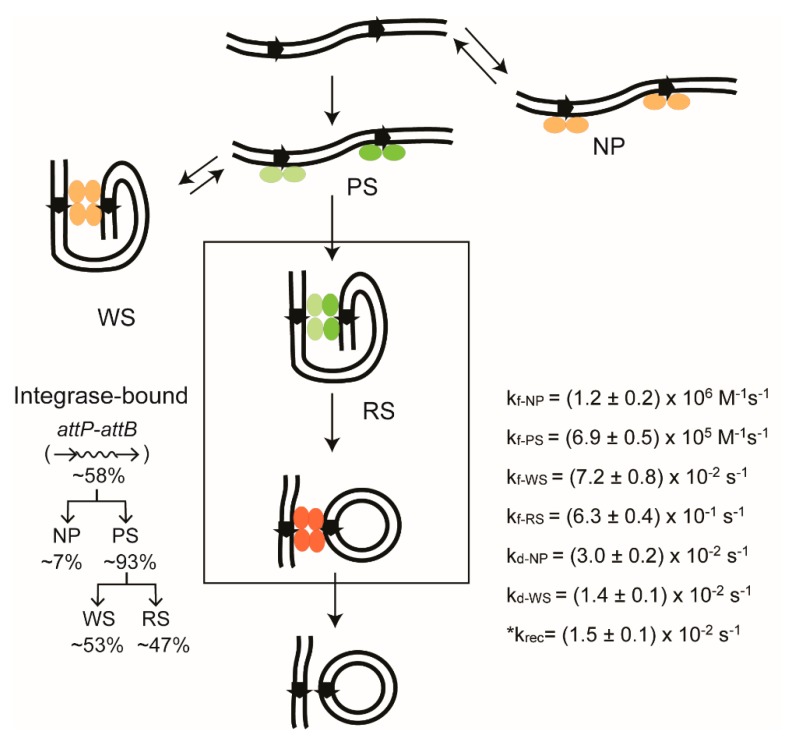
TPM picture of recombination by ϕC31 integrase from start to finish. The diagram is arranged as in Figure 7, and depicts the reaction between head-to-tail attP–attB sites by the integrase in the absence of the RDF protein. The productive and abortive integrase–DNA complexes are named as in Figure 7. The integrase dimers in the functional reaction path are shown in green, and those external to this path are shown in pale orange. In the product synapse containing attL and attR, the dimers are shown in orange to indicate that attL–attR recombination does not occur in the absence of RDF. The relative abundance of individual complexes is indicated, and the kinetic constants for their transitions are listed. There is no Holliday junction intermediate in this reaction. The attP–attB reaction is blocked in the presence of RDF in amounts sufficient to channel the synapse quantitatively into its inactive configuration. Similar reactions schemes with kinetic parameters have been derived for inversion between head-to-head attP–attB sites promoted by the integrase, and for deletion between head-to-tail attL–attR sites promoted by integrase plus RDF. The details can be found in Fan et al. [43]. The rate constant for recombination k_rec_, marked by an asterisk, was derived from the inversion reaction between attP–attB sites in head-to-head orientation.

We analyzed the individual steps of the attP × attB and attL × attR reactions [43] using DNA substrates and experimental procedures analogous to those employed for studying Cre and Flp recombination [41,44]. We observed the formation of pre-synaptic complexes and non-productive complexes by integrase binding to attP–attB, as well as the conversion of pre-synaptic complexes to synaptic complexes that complete recombination, or to wayward complexes that synapse abortively and dissociate. The TPM time traces for individual molecules permitted us to quantitate these events and to assign kinetic constants to the forward and reverse transitions of individual complexes (Figure 10). Similar analyses were also performed on attL–attR substrates in the presence and absence of RDF. Our cumulative results are consistent with RDF regulating the directionality of recombination by selectively promoting or blocking the active conformations of the synaptic structures formed by specific att site partners. Our findings support a ‘gated rotation’ model [43,48,76], in which the cleaved strands are joined after a relative rotation of 180° (attP × attB giving attL and attR, and vice versa), effectively reducing the chances for multiple rounds of rotation of the cleaved DNA. This observation is somewhat at odds with the conclusion from the prior single molecule study of Bxb1 integrase; that the recombination synapse remains rotationally open for a long time [46]—several minutes, in fact.

## 3. Summary and Perspective

TPM has proven to be a powerful method for incisively probing the mechanistic features of DNA–protein interactions in a variety of biological contexts. We have taken advantage of TPM to understand the mechanism of action of site-specific recombinases—initially the tyrosine recombinases, Cre and Flp, and subsequently the serine recombinase, ϕC31 integrase. In conjunction with relatively low or large force fields induced by flow or applied using optical/magnetic tweezers, respectively, TPM can be adapted to address specialized areas of DNA–protein interactions—protein mediated compaction or flexibility changes in DNA induced by architectural proteins, bridging of distant DNA sites and DNA looping mediated by proteins that regulate gene expression, and translocation of protein machines along DNA. TFM and TFM-FRET are variations of the principle of TPM, whereby relatively long-range conformational changes in DNA are followed by the movement of a single fluorophore (TFM), and relatively short-range conformational transitions are probed by the movement of a fluorophore pair (TFM-FRET). Multiplex TPM can facilitate high-throughput analyses of a variety of DNA–protein interactions. Potential further advances are likely to follow with improvements to 3D-TPM and its increased application.

We anticipate future progress in our understanding of site-specific recombination through continued use of TPM. As noted earlier, a subset of the serine recombinases—the Tn3 resolvase, for example—are difficult to study by TPM because of their strict requirement of supercoiled substrates. However, point mutations in these enzymes that free them from supercoiling requirements have been characterized [77,78]. These self-activated recombinase variants can be readily analyzed by standard TPM methodology. An RDF-independent mutant of ϕC31 integrase, Int(E449K), which can catalyze attL–attR recombination by itself [79], is available. Furthermore, a fusion protein between the integrase and RDF has been shown to follow the same regulation of recombination directionality as that established by RDF added in trans to the wild type integrase [80]. Analysis of the RDF-independent integrase and the hybrid integrase-RDF by TPM would be useful in providing additional insights into the specific steps of recombination controlled by RDF.
